# Genomic characterization of a new endophytic *Streptomyces kebangsaanensis* identifies biosynthetic pathway gene clusters for novel phenazine antibiotic production

**DOI:** 10.7717/peerj.3738

**Published:** 2017-11-29

**Authors:** Juwairiah Remali, Nurul ‘Izzah Mohd Sarmin, Chyan Leong Ng, John J.L. Tiong, Wan M. Aizat, Loke Kok Keong, Noraziah Mohamad Zin

**Affiliations:** 1 School of Diagnostic and Applied Health Sciences, Faculty of Health Sciences, Universiti Kebangsaan Malaysia, Kuala Lumpur, Malaysia; 2 Centre of PreClinical Science Studies, Faculty of Dentistry, Universiti Teknologi MARA Sungai Buloh Campus, Sungai Buloh, Selangor, Malaysia; 3 Institute of Systems Biology (INBIOSIS), Universiti Kebangsaan Malaysia, Bangi, Selangor, Malaysia; 4 School of Pharmacy, Taylor’s University, Subang Jaya, Selangor, Malaysia

**Keywords:** *S. kebangsaanensis*, Phenazine, Secondary metabolites, Genomic

## Abstract

**Background:**

*Streptomyces* are well known for their capability to produce many bioactive secondary metabolites with medical and industrial importance. Here we report a novel bioactive phenazine compound, 6-((2-hydroxy-4-methoxyphenoxy) carbonyl) phenazine-1-carboxylic acid (HCPCA) extracted from *Streptomyces kebangsaanensis*, an endophyte isolated from the ethnomedicinal *Portulaca oleracea.*

**Methods:**

The HCPCA chemical structure was determined using nuclear magnetic resonance spectroscopy. We conducted whole genome sequencing for the identification of the gene cluster(s) believed to be responsible for phenazine biosynthesis in order to map its corresponding pathway, in addition to bioinformatics analysis to assess the potential of *S. kebangsaanensis* in producing other useful secondary metabolites.

**Results:**

The *S. kebangsaanensis* genome comprises an 8,328,719 bp linear chromosome with high GC content (71.35%) consisting of 12 rRNA operons, 81 tRNA, and 7,558 protein coding genes. We identified 24 gene clusters involved in polyketide, nonribosomal peptide, terpene, bacteriocin, and siderophore biosynthesis, as well as a gene cluster predicted to be responsible for phenazine biosynthesis.

**Discussion:**

The HCPCA phenazine structure was hypothesized to derive from the combination of two biosynthetic pathways, phenazine-1,6-dicarboxylic acid and 4-methoxybenzene-1,2-diol, originated from the shikimic acid pathway. The identification of a biosynthesis pathway gene cluster for phenazine antibiotics might facilitate future genetic engineering design of new synthetic phenazine antibiotics. Additionally, these findings confirm the potential of *S. kebangsaanensis* for producing various antibiotics and secondary metabolites.

## Introduction

*Streptomyces* are Gram positive, filamentous saprophytes known for their roles in producing various secondary metabolites important for medicinal therapies (Hopwood, 2007; Kieser, 2000). Despite the continuous efforts to isolate novel drug compounds from soil-dwelling *Streptomyces,* the numbers of newly identified compounds have been dwindling over the years (Palaez, 2006). One strategy to increase the chances of identifying new bioactive compounds as well as to combat the scourge of antimicrobial resistance is to investigate microorganism sources. Recently, it has since become clear that some *Streptomyces* sp. also exist as endophytes that dwell within the tissues of certain plants (Castillo et al., 2006; Ezra et al., 2004; Strobel & Daisy, 2003). The possibility that this unique living environment of endophytes may be the niche of many other unidentified species or strains of bacteria has gained our attention for its potential in unravelling new sources of biologically active compounds with industrial or medicinal applications (Ghadin et al., 2008; Strobel & Daisy, 2003).

In particular, *Streptomyces kebangsaanensis* represents a novel endophyte isolated from the ethnomedical plant, *Portulaca oleracea* Linn, known in Malaysia as ‘Gelang pasir’, that was demonstrated to have medicinal and pharmaceutical properties such as antiseptic and anti-inflammatory activities (Lim & Quah, 2007; Sarmin et al., 2013). When cultured on International *Streptomyces* Project 2 agar, the formation of greenish-yellow substrate mycelia and greenish-grey aerial hyphae were readily visible (Sarmin et al., 2013). *S. kebangsaanensis* (*Streptomyces* SUK12^T^; GenBank accession number: HM449824) is a Gram-positive bacterium (Family: Streptomycetaceae; Class: Actinobacteria) (Sarmin et al., 2013). Recently, it has been found to produce the bioactive compound phenazine-1-carboxylic acid (known as tubermycin B), which was shown to have antibacterial, anticancer, antiparasitic, and antiviral properties (Laursen & Nielsen, 2004; Sarmin et al., 2013). This suggests that *S. kebangsaanensis* represents an untapped source of bioactive compounds such as phenazines that might be potentially further utilised. However, more studies are needed to characterise other bioactive compounds from this species as well as to elucidate the genes responsible for the biosynthesis of these metabolites.

Phenazines are a group of nitrogen-containing heterocyclic compounds known for their antibacterial, antifungal, antiviral, and anticancer functions (Laursen & Nielsen, 2004; McDonald et al., 2001). These compounds are derived from bacteria of diverse genera such as *Pseudomonas*, *Streptomyces*, *Vibrio and Pelagiobacter* (Mavrodi et al., 2010). Notably, while more complex phenazines are biosynthesized by *Streptomyces,* less complex derivatives are normally obtained from *Pseudomonas* (Laursen & Nielsen, 2004). The first known phenazine isolated from *Streptomyces* was the antibiotic griseolutien (Umezawa et al., 1950) and subsequently many *Streptomyces* sp. have been shown to produce numerous diverse and complex phenazines including lomofungin from *Streptomyces lomondensis* (Johnson & Dietz, 1969) and endophenazines from *Streptomyces anulatus* (Gebhardt et al., 2002; Krastel et al., 2002). Although the phenazine biosynthesis core structure has already been described (Haagen et al., 2006; Mavrodi et al., 1998), the formation of more complex phenazine structures are still hypothetical and largely unknown (Mentel et al., 2009). Therefore, more research in elucidating the biosynthetic pathway of complex phenazines from *Streptomyces* species such as *S. kebangsaanensis* is crucial.

In the current study, we successfully isolated a novel phenazine compound termed 6-((2-hydroxy-4-metoxyphenoxy) carbonyl) phenazine-1-carboxylic acid (HCPCA) from *S. kebangsaanensis*. Its molecular structure was elucidated using nuclear magnetic resonance spectroscopy (NMR). This structure was then compared against other known phenazine compounds available in public databases to search for closely related compounds.

In order to discover the biosynthesis of this novel compound as well as other metabolites from *S. kebangsaanensis*, genome sequencing was carried out using IIlumina Hiseq2000. Several gene clusters including a phenazine biosynthetic gene cluster were identified and used to further elucidate the biosynthesis pathway of HCPCA.

## Material and Methods

### Secondary metabolite extraction and isolation of HCPCA from *S. kebangsaanensis*

The crude extract from *S. kebangsaanensis* was obtained using a modified protocol detailed in (Zin et al., 2007). Briefly, the bacteria isolate was subcultured on Bn-2 agar and incubated at room temperature (RT) (28–30 °C) for 14 days. Then, five blocks of agar (1 cm × 1 cm) of matured *S. kebangsaanensis* were added into 200 mL of V22 broth as seeding culture. The broth was incubated for four days at RT with gentle shaking (140 rpm) using an orbital shaker. Subsequently, 3% of the seeding culture was inoculated into fermentation media (A_3_M) that was supplemented with resin. The broth was then agitated (140 rpm) and incubated for 10 days. Three and a half volumes of acetone were used to extract the culture filtrates. The pooled organic phase was subsequently dried using a Rotavapor (Eyela Rotary Vacuum Evaporator N-N series; Eyela, Tokyo, Japan) at 40 °C. This crude extract was then weighed, fractionated, and isolated to be further analysed.

The crude samples were separated using vacuum liquid chromatography, radial chromatography (RC), and preparative thin layer chromatography (TLC) (Fig. S1). After each chromatography step, an antimicrobial assay against *B. subtilis* ATCC 6633 was performed on each fraction to determine its activity for subsequent isolation (Sarmin, 2012). Briefly, a total of 32.15 g acetone crude extract was separated into six fractions by using vacuum liquid chromatography with hexane: chloroform (8:2); hexane:chloroform (6:4); chloroform 100%, and 100% methanol solvent systems. The active fraction 5 (F5) was separated again into four sub-fractions using RC with the hexane:chloroform (9:1) solvent system. Subsequently, the active sub-fraction 3 (F3) was further fractionated using RC with the hexane:chloroform (2:8) solvent system, which produced seven sub-fractions. The active sub-fraction 7 (F7) was then separated to four more sub-fractions using RC with the hexane:chloroform:methanol (7:2:1) solvent system from which an active sub-fraction 1 (F1) was obtained. This sub-fraction was then isolated via preparative TLC using a hexane:ethyl acetate (3:7) solvent system, which produced four sub-fractions. Sub-fraction 1 was purified using an Agilent 1200 HPLC system (Santa Clara, CA, USA) equipped with a C-18 column (4.6 × 250 mm, 5 µm) and the mobile phase was made up of 0.1% trifluoroacetic acid added to 5% methanol:95% acetonitrile. A gradient elution step was employed as shown in Table S2. These techniques successfully purified an active compound termed AF53611 (0.5 mg) (Fig. S1).

### Antibiotic resistance profile

The ability of strain SUK 12 to grow in the presence of antibiotic was tested against vancomycin, gentamicin, ampicillin, penicillin G, amphotericin B, tetracyclin, streptomycin, methicillin, cyclohexamide, oxacillin, nystatin dan nalidixic acid. Suspension of bacterial culture was set at 0.1 optical density at 625 nm wavelength using spectrophotometer (SECOMAM). Then, the suspension was lawn on International Streptomyces Agar 2 (ISP2). After allowing the suspension to absorb into the agar (1 min), the antibiotic disc (6 mm) were placed evenly on the surface of the plate with a sterile forcep. Plates were then incubated for 3–5 days at 28 °C. The antibiotics resistance profile was shown in Table S1.

### Structure determination of HCPCA using NMR

The isolated pure AF53611 compound was dissolved in deuterated methanol prior to submission to an NMR facility (Bruker 600 MHz FT-NMR) at the School of Chemical Sciences & Food Technology, Faculty of Science and Technology, Universiti Kebangsaan Malaysia. The tests utilized consisted of one dimensional (^1^HNMR and ^13^C-APT) and two dimensional (^1^H-^1^H COSY and ^1^H-^13^C HMBC) techniques. The structure obtained was then compared with other known phenazine compounds from the NCBI database (http://www.ncbi.nlm.nih.gov/pcsubstance/?term=phenazine; accessed October 13, 2016).

### Whole genome sequencing

The *S. kebangsaanensis* strain was obtained from the stock culture of the Novel Antibiotic Research Laboratory, UKM. Genomic DNA extraction was performed following (Kieser, 2000) with slight modifications. *S. kebangsaanensis* genome sequencing was carried out at the Malaysian Genomic Resource Centre (MGRC), Mid Valley, Malaysia. The sequencing procedures were as follows. Genomic DNA was fragmented (400–600 bp) using a Covaris S220 focused ultrasonicator (Covaris Inc., Wolburn, MA, USA). The DNA fragments were then end-repaired before ligated to Illumina TruSeq adapters. The DNA was further enriched using the TruSeq DNA Sample Preparation Kit (Illumina, San Diego, CA, USA) according to the manufacturer’s protocol. The quantification of the final sequencing library was carried out using a KAPA kit (KAPA Biosystems, Wilmington, MA, USA) on an Agilent Stratagene Mx-3005p qPCR machine and library size was validated using Agilent Bioanalyzer High Sensitivity DNA Chip. The sequencing of the whole genome was carried out using an Illumina Genome Analyzer based on the manufacturer’s instructions. The reads were first filtered and assembled into contigs using the *in-house* assembler pipeline called SynaDNovo. Contigs were further assembled using paired-end library information to form scaffolds. The annotation was accomplished using MGRC pipeline, SynaSearch, and Rapid Annotation Using Subsystem Technology (RAST) (Aziz et al., 2008). The data from this whole genome shotgun project were deposited at DDBJ/EMBL/GenBank under BioProject; PRJNA269542 and BioSample; SAMN03254380.

### Bioinformatics analysis

The tRNA and rRNA genes were predicted using ARAGORN (Laslett & Canback, 2004) and rRNAmmer (Lagesen et al., 2007). Subsequently, antiSMASH 3.0 was employed to identify genes encoding secondary metabolites (Medema et al., 2011), with rapid identification of a whole range of known secondary metabolite compound classes. The phenazine biosynthetic pathway was manually constructed and cross-checked with cited published reviews/papers. The BLAST analysis of the putative phenazine gene cluster against other genomes was performed using MUMmer 3.0 (Kurtz et al., 2004). The image of the putative operon against genomes was produced using the BLAST Ring Image Generator (BRIG) (Alikhan et al., 2011). Gene ontologies were analysed and plotted using BGI Wego (Ye et al., 2006) and the corresponding phylogenetic tree was developed using MEGA4 (Tamura et al., 2007).

## Results

### Isolation and structural determination of HCPCA

**Figure 1:**
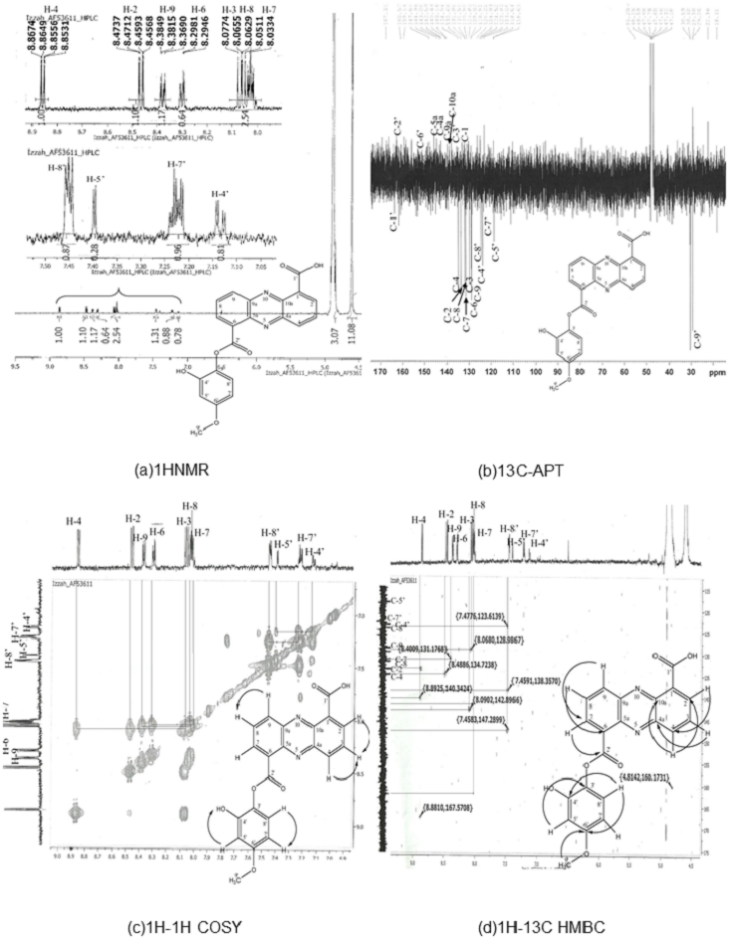
Structure determination for compound AF53611, later discovered as 6-((2-hydroxy-4-metoxyphenoxy) carbonyl) phenazine-1-carboxylic acid (HCPCA). The structure was elucidated using one dimensional (1HNMR and 13C-APT) (A and B) and two dimensional (1H-1H COSY and 1H-13C HMBC) (C and D) techniques.

Results of ^1^HNMR, ^13^C-APT^1^H-^1^H correlation spectroscopy (COSY), and ^1^H-^13^C heteronuclear multiple bond coherence (HMBC) are shown in Fig. 1. C_21_H_14_N_2_O_6_ (390), UV in MeOH, *λ*max/nm (log ε): 246, 363.8; EI-MS m/z (relative intensity) 390 (26.4, Na^+^); ^1^H-NMR (MeOD, 600 MHz) *δ* 8.86 (1 H, dd, *J* = 7:08, 1.5, H-4), 8.47 (1 H, dd, *J* = 8.64, 1.5, H-2), 8:08 (1 H, m, H-3), 8.03 (1 H, m, H-7), 8.06 (1 H, m, H-8), 8.38 (1 H, m, H-9), 15.61 (1 H, s, 1′-OOH), 7.14 (1 H, m, 4′-OH), 7:40 (1 H, m, H-5′), 7.22 (1 H, m, H-7′), 7.45 (1 H, m, H-8′), and 1.34 (3 H, s, H-9′). ^13^C NMR (MeOD, 600 MHz) detected 21 carbon signals. We observed 9 carbon methines that were absorbed at δ 133.68 (C-2), 129.83 (C-3), 134.94 (C-4), 131.51 (C-7), 131.96 (C-8), 128.91 (C-9), 118.84 (C5), 123.61 (C7) and 124.52 (C-8′). The carbon quaternary signals were absorbed at δ 126.22 (C-1), 142.50 (C-4a), 143.06 (C-5a), 129.02 (C-6), 140.49 (C-9a), 140.18 (C-10A), and 138.38 (C-3′). The aromatic quaternary carbon signal was absorbed at δ 163.90 (C-1′) whereas the methyl carbon signal was absorbed at δ 29.63 (C-9) (Table S3). The obtained structure (Fig. 1) exhibits a phenazine core structure with additional functional groups.

This HCPCA structure was then compared against all known phenazine structures in the NCBI database without detecting any similarities. The most closely related compound was saphenamycin with an additional methyl group and a different functional group [6-(1-hydroxyethyl)1-phenazinecarboxylic acid instead of 4-methoxybenzene-1,2-diol in HCPCA] (Fig. S2).

### Genomic Study of *S. kebangsaanensis*

To elucidate the biosynthetic genes and related pathways of phenazines in *S. kebangsaanensis*, whole genome sequencing followed by bioinformatics analysis was performed. Whole genome sequencing using HiSeq2000 (Illumina, San Diego, CA, USA) resulted in 2.6 Gbp raw reads. Reads pre-processing was performed to remove adaptors as well as low quality and ambiguous bases. The sequences were then assembled using *de-novo* assembly, which produced 560 contigs and 170 scaffolds. The longest scaffold contained 453,879 base pair (bp) whereas the shortest was 1,072 bp, with the median (N50) and mean length being 110,454 bp and 48,992 bp, respectively (Table 1). The draft genome sequence of *S. kebangsaanensis* consisted of 8,328,719 bp, with an average GC content of 71.35% (Table 1). Gene sequence annotation predicted 8,001 open reading frames including 7,558 genes with known function; the remainder (443 genes) did not result in any significant BLAST hits (Table 2). The *S. kebangsaanensis* genome also contained high numbers of tRNA gene sequences (80), one sequence of tmRNA, and 12 operons of rRNA (16S-23S-5S), which was comparable to other *Streptomyces* (Table 2).

**Table 1:** Genomic data for *Streptomyces kebangsaanensis*.

Assembled scaffolds	170
Predicted gene	8,001
Total size	8,328,719
Longest	453,879
Shortest	1,072
N50	110,454
Mean size	48,992
% A	14.16
% C	35.58
% G	35.77
% T	14.19
% N	0.30

**Table 2:** Genomic feature comparison of *S. kebangsaanensis* with other *Streptomyces*.

No.	Species	Length (Mbp)	Avg. G + C content (%)	No. of protein coding genes	No. of rRNA (16S-23S-5S)	No. of tRNA genes	No. of other RNAs	References
1.	*Streptomyces kebangsaanensis*	8.32	71.35	7558	12	80	1	–
2.	*Streptomyces griseus*	8.54	72.2	7138	6	66	1	Ohnishi et al. (2008)
3.	*Streptomyces coelicolor* A3(2)	8. 67	72.2	7825	6	63	1	Bentley et al. (2002)
4.	*Streptomyces avertimilis*	9.03	70.7	7583	6	68	1	Ōmura et al. (2001)
5.	*Streptomyces albus* J1074	6.84	73.3	5746	21	66	1	Zaburannyi et al. (2014)
6.	*Streptomyces bingchenggensis* BCW-1	11.94	70.8	9309	18	66	3	Wang et al. (2010)
7.	*Streptomyces cattleya* NRRL 8057	6.28	72.9	5360	18	64	1	Barbe et al. (2011)
8.	*Streptomyces davawensis* JCM4913	9.47	70.6	8174	18	70	3	Jankowitsch et al. (2012)
9.	*Streptomyces fulvissimus* DSM40593	7.91	71.5	6729	18	73	3	Myronovskyi et al. (2013)
10.	*Streptomyces hygroscopicus subsp jingangensis* 5008	10.15	71.9	8673	18	70	5	Wu et al. (2012)
11.	*Streptomyces scabiei* 87.22	10.15	71.5	8440	18	75	3	Yaxley (2009)
12.	*Streptomyces* sp*.* PAMC 26508	7.53	71.06	6345	18	68	3	Data deposited in NCBI without publication
13.	*Streptomyces sp.* Sirex AA-E	7.41	71.7	6331	19	64	3	Data deposited in NCBI without publication
14.	*Streptomyces venezuelae* ATCC 10712	8.23	72.4	7080	12	66	1	Pullan et al. (2011)
15.	*Streptomyces violacuesniger* Tu 4113	10.66	71.0	8264	18	64	3	Data deposited in NCBI without publication

**Figure 2:**
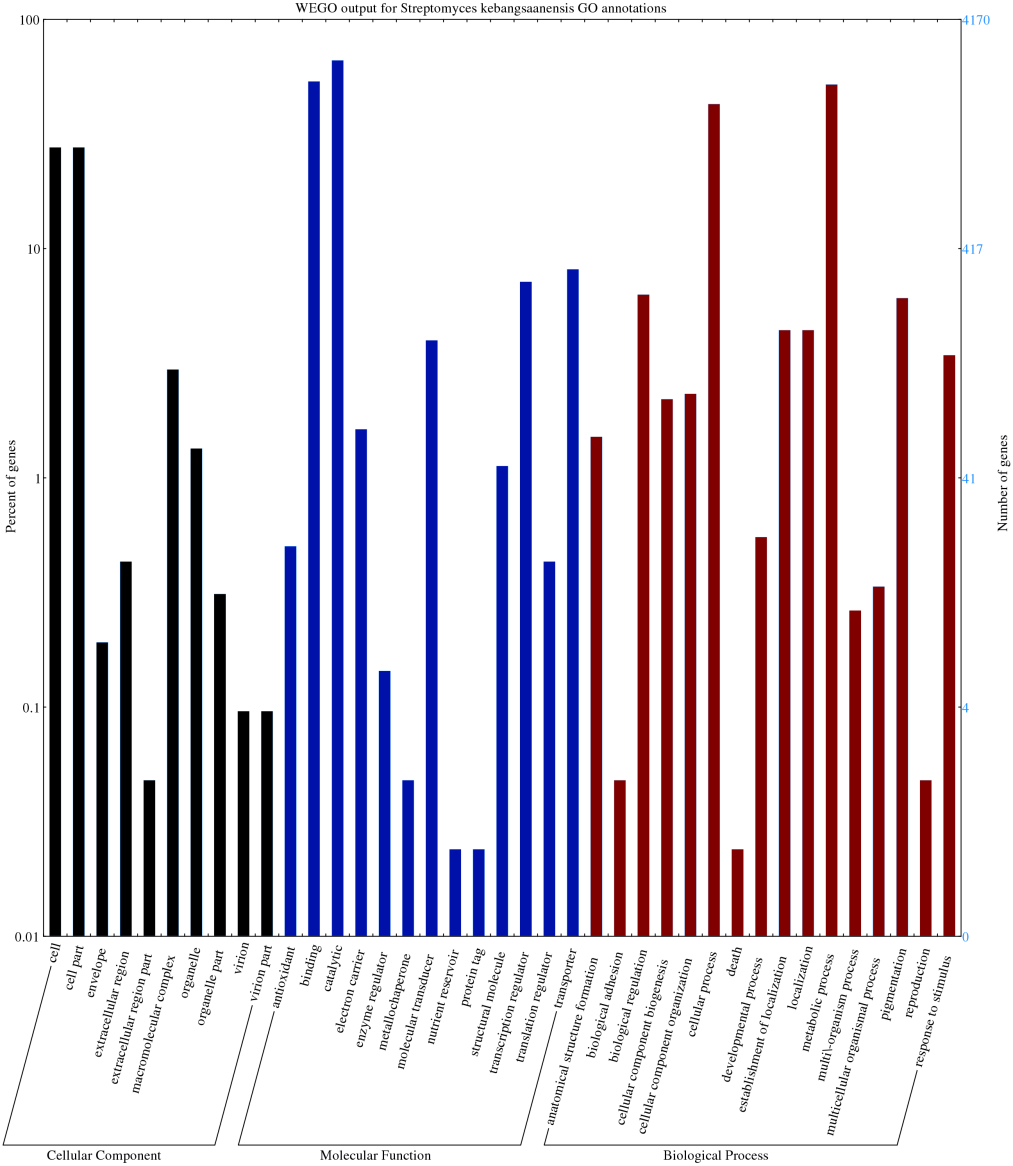
The gene ontology of *Streptomyces kebangsaanensis* which has been classified into cellular component, molecular function and biological process categories. Cell and cell part contribute to the highest percentage of genes in the cellular component category of *S. kebangsaanensis* genome. In molecular function, the genes that are involved in catalytic activity, binding and transportation account for the highest percentage of genes in this class. Meanwhile, more than 60% of the genes are involved in metabolic process of *S. kebangsaanensis* within the category of biological process. Secondary metabolite genes are classified in the response to stimulus (biological process category).

The predicted genes/open reading frames were functionally categorized using Gene Ontology (GO) annotations (Consortium, 2013) of which 3,238 genes were predicted to be involved in numerous biological processes, 1,402 genes in cell components, and 6551 in molecular functions (Fig. 2). The neighbour-joining phylogenetic tree generated based on 16S rRNA gene sequences (1,599 nt) specified the evolutionary relationship between *S. kebangsaanensis* with other members of the *Streptomyces* (Fig. S3).

**Figure 3:**
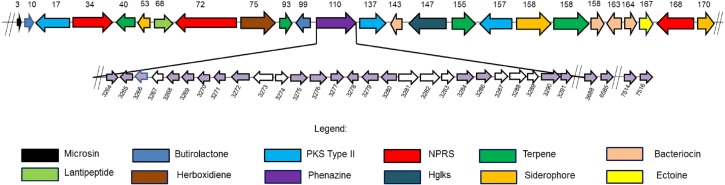
Twenty four secondary metabolite gene clusters predicted in *S. kebangsaanensis.* These gene clusters have been predicted using antiSMASH (except the phenazine biosynthetic gene cluster at scaffold 110 that was manually identified). Each colour of gene represents a different class of antibiotics and secondary metabolites, as shown in the legend. Each secondary metabolite gene cluster located in different scaffold of *S. kebangsaanensis* (total scaffold in the genome is 170) as shown in the numbering above the gene cluster. The genes have been ordered based on the location of predicted secondary metabolite gene cluster in the different scaffold (split loci). Phenazine biosynthetic gene cluster is located at scaffold 110 in the genome of *S. kebangsaanensis*. The light purple arrows represent known genes that are involved in the phenazine biosynthesis whereas the white arrows represent unknown genes that may be involved in the pathway. The numbering below the gene clusters are the location of each gene in the genome of *S. kebangsaanensis* (from the total of 8,001 genes based on the identified open reading frame). The forward arrows represent the forward genes whereas the reverse arrows represent the reverse genes.

### Antibiotic and secondary metabolite gene clusters

The analysis of the *S. kebangsaanensis* genome using Antibiotics & Secondary Metabolite Analysis SHell (antiSMASH) (Medema et al., 2011) software led to the identification of 24 biosynthetic gene clusters from among the 170 identified scaffolds with most being responsible for antibiotic and other secondary metabolites production (Fig. 3). These gene clusters were mainly involved in terpene and bacteriocin biosynthesis, followed by the biosynthesis of siderophores, nonribosomal peptide-synthase (NRPS) enzymes, polyketide synthase (PKS) type II, lantipeptide, and butyrolactone (Fig. 3). In particular, *S. kebangsaanensis* was predicted to produce at least four terpenes, with their corresponding gene clusters located at scaffolds 40, 93, 155, and 158 (Fig. 3). In addition, the genome of *S. kebangsaanensis* contained four gene clusters for the biosynthesis of bacteriocin as well as three clusters of genes each for siderophore, PKS type II and NRPS production (Fig. 3). Furthermore, butyrolactone was associated with two biosynthetic gene clusters, whereas PKS type III, lantipeptide, and ectoine each matched only one gene cluster (Fig. 3).

Notably, the antiSMASH software used in the current study represents, to our knowledge, the only software package that can detect the entirety of secondary metabolite gene clusters in microbial genomes (Fedorova, Moktali & Medema, 2012; Medema et al., 2011). antiSMASH is a comprehensive platform used for the identification of gene clusters encoding enzymes responsible for the production of various secondary metabolites (Medema et al., 2011) and was successfully utilized in this study to identify the 24 gene clusters described above. However, antiSMASH was not able to classify the phenazine biosynthetic gene cluster in *S. kebangsaanensis*. This might be due to discrepancies in the antiSMASH database as its phenazine gene cluster reference was mainly derived from *Pseudomonas* sp. instead of the more complex *Streptomyces* sp. clusters (Laursen & Nielsen, 2004). Other programs such as CLUSEAN, NRPSPredictor, and SBSPKS were more suitable for specifically detecting NRPS and PKS genes but not those of other classes of secondary metabolites including phenazines (Fedorova, Moktali & Medema, 2012). Therefore, the phenazine genes and their corresponding clusters were manually constructed and cross-checked with several other cited published reviews/papers.

**Table 3:** Putative gene clusters that were predicted to involve in the phenazine biosynthesis of *Streptomyces kebangsaanensis.*

No	Gene	Nucleotide (nt)	Location in genome	Strand	Protein	Organisms	Identity	*E*-value	Bit score	Accession number
1.	gene_3264	489	7160–7648	+	Transcriptional regulator, MarR family	*Catenulisporaacidiphila*	37.81%	2.00E−24	103	C7QAG0
2.	gene_3265	1101	7760–8860	–	Transposase	*Streptomyces* sp. CNQ-418	54.85%	2.00E−119	363	J7H1A5
3.	gene_3266	1023	9287–10309	–	Putative oxidoreductase	*Gordonia rhizosphere* NBRC 16068	45.57%	9.00E−76	248	K6V427
4.	gene_3267	888	10311–11198	–	N-acetyltransferase	*Streptosporangiumroseum*	43.87%	1.00E−55	191	D2AUQ7
5.	gene_3268	1014	11213–12226	–	Putative 3-oxoacyl-[acyl-carrier-protein] synthase III	*Kitasatospora setae* ATCC 33774	50%	3.00E−97	302	E4N0J5
6.	gene_3269	1953	12223–14175	–	Putative anthranilate synthase	*Streptomyces fulvissimus* DSM 40593	67%	0	593	N0CTI1
7.	gene_3270	687	14172–14858	–	Phenazine biosynthesis protein D	*Streptomyces gancidicus* BKS 13-15	69%	6.00E−99	298	M3BLH5
8.	gene_3271	816	14891–15706	–	2,3-dihydroxybenzoate-2,3-dehydrogenase	*Streptomyces gancidicus* BKS 13-15	68%	1.00E−94	291	M3CKN3
9.	gene_3272	1173	15745–16917	–	3-deoxy-7-phosphoheptulonate synthase	*Streptomyces fulvissimus* DSM 40593	68%	5.00E−173	500	N0CZ35
10.	gene_3273	1614	17034–18647	–	Putative carboxylesterase	*Streptomecesfulvissimus* DSM 40593	58%	5.00E−180	530	N0CXC2
11.	gene_3274	705	19134–19838	+	N-acetyltransferase	*Streptomyces fulvissimus* DSM 40593	47%	8.00E−64	211	N0CRH9
12.	gene_3275	1581	19987–21567	+	Acyl-CoA synthetase	*Streptomyces silvensis*	77%	0	782	E4N0H6
13.	gene_3276	1569	21564–23132	+	Putative monooxygenase	*Kitasatospora setae* ATCC 33774	61%	0	557	E4N0J7
14.	gene_3277	564	23181–23844	+	Multimeric flavodoxinWrbA	*Streptomyces venezuela* ATCC 10712	74%	2.00E−84	259	F2R5F0
15.	gene_3278	549	24141–24689	–	Polyketide cyclase	*Streptomyces aureocirculatus*	69%	1.00E−75	233	L7PIK0
16.	gene_3279	1185	24762–25946	–	Salicyclatehyroxylase	*Streptomyces sp. NRRL S-337*	72%	2.00E−177	508	D6AXN4
17.	gene_3280	1014	25999–27012	–	Putative oxidoreductase	*Streptomyces hygroscopicus* subsp. *jinggangensis* TL01	71%	4.00E−128	381	M1MB75
18.	gene_3281	1944	27262–29205	+	Putative uncharacterized protein	*Streptomyces viridochromogenes* DSM 40736	74.45%	0	937	D9XDR3
19.	gene_3282	1845	29202–31046	+	3-carboxy-cis,cis-muconate cycloisomerase	*Streptomyces* sp. HPH0547	70.72%	2.00E−151	457	S3B8V3
20.	gene_3283	681	31224–31904	+	Hemerythrin HHE cation binding domain protein	*Frankia symbiont* subsp. *Datiscaglomerata*	47.76%	3.00E−44	158	F8B4H0
21.	gene_3284	1107	32030–33136	+	Putative oxidoreductase	*Streptomyces scabies* (strain 87.22)	46.13%	6.00E−76	251	C9Z6J1
22.	gene_3286	861	33943–34803	+	3-oxoacyl-(Acyl-carrier-protein) synthase 3	*Streptomyces bingchenggensis* BCW-1	44%	7.00E−66	219	D7C4C2
23.	gene_3287	762	34990–35751	+	3-hydroxyacyl CoA dehydrogenase	*Amycolatopsismediterranei* U-32	61.88%	5.00E−57	193	D8HNP0
24.	gene_3288	1026	35765–36790	+	Putative F420 dependent oxidase	*Streptomyces flavogriseus* ATCC 33331/ DSM 40990/ IAF-45CD	76.25%	3.00E−155	451	E8WAF8
25.	gene_3289	117	36787–36963	+	NAD binding protein 3-hydroxylacyl-CoA dehydrogenase	*Streptomyces mobaraensis* NBRC 13819=DSM 40847	79.49	3.00E−11	67	M3BJ16
26.	gene_3290	1212	37080–39291	+	2-component transcriptional regulator	*Streptomyces pristinaespiralis* ATCC 25486	85.79%	8.00E−158	469	B5HHN5
27.	gene_3291	687	38408–39094	+	Regulatory protein	*Streptomyces pristinaespiralis*	90.99%	1.00E−138	402	B5HHN6
28.	gene_7514	1353	376052–377404	–	2-dehydro-3-deoxyphosphoheptonate aldolase (C)	*Streptomyces ghanaensis* ATCC 14672	97.98%	0	915	D6A8C0
29.	gene_7516	1857	379032–380888	+	Putative anthranilate synthase, phenazine specific (E)	*Streptomyces afghaniensis* 772	89%	0	880	S4MTI6
30.	gene_6585	645	85431–86075	–	Phenazine biosynthesis C/F protein	*Streptomyces lividans* TK24	89%	4.00E−120	351	D6EHD0
31.	gene_3688	813	34414–35226	–	Phenazine biosynthesis protein F	*Streptomyces collinus* Tu365	86%	2.00E−168	479	S1SF07

### Phenazine genes and predicted phenazine gene clusters

We hypothesized that 31 genes might be responsible for phenazine biosynthesis in *S. kebangsaanensis*. These include phenazine modification genes, resistance genes, as well as regulatory genes (Table 3). The majority of these genes (27) were located in scaffold 110 including genes for the phenazine core structure (putative anthranilate synthase, *phz*E; phenazine biosynthesis protein, *phz*D; 2,3-dihydroxybenzoate-2,3-dehydrogenase, *phz*A; and 3-deoxy-7-phosphoheptulonate synthase, *phz*C) and were therefore referred to as the phenazine cluster. Notably, other predicted phenazine genes were also found located in scaffold 120 (phenazine biosynthesis protein; *phz*F), scaffold 163 (phenazine biosynthesis *phz*C/*phz*F protein), and scaffold 167 (2-dehydro-3-deoxyphosphoheptonate aldolase, *phz*C; and putative anthranilate synthase, *phz*E) (Table 3). In comparison, the *Streptomyces cinnamonensis* DSM1042 genome also showed a slightly similar gene distribution profile, wherein all the genes essential for phenazine biosynthesis were found to be located within two different loci (Seeger et al., 2011).

However, an additional nine genes were present in the phenazine cluster that were determined by exhaustive comparison against other previous studies as not having been previously annotated as representing phenazine biosynthetic genes. These genes were identified as N-acetyltransferases (two genes), putative carboxylesterase, 3-carboxy-cis,cis-muconate cycloisomerase, hemerythrin HHE cation binding domain protein, 3-hydroxyacyl CoA dehydrogenase, putative F420 dependent oxidase, NAD binding protein 3-hydroxylacyl-CoA dehydrogenase, and a putative uncharacterized protein (Table 3).

**Figure 4:**
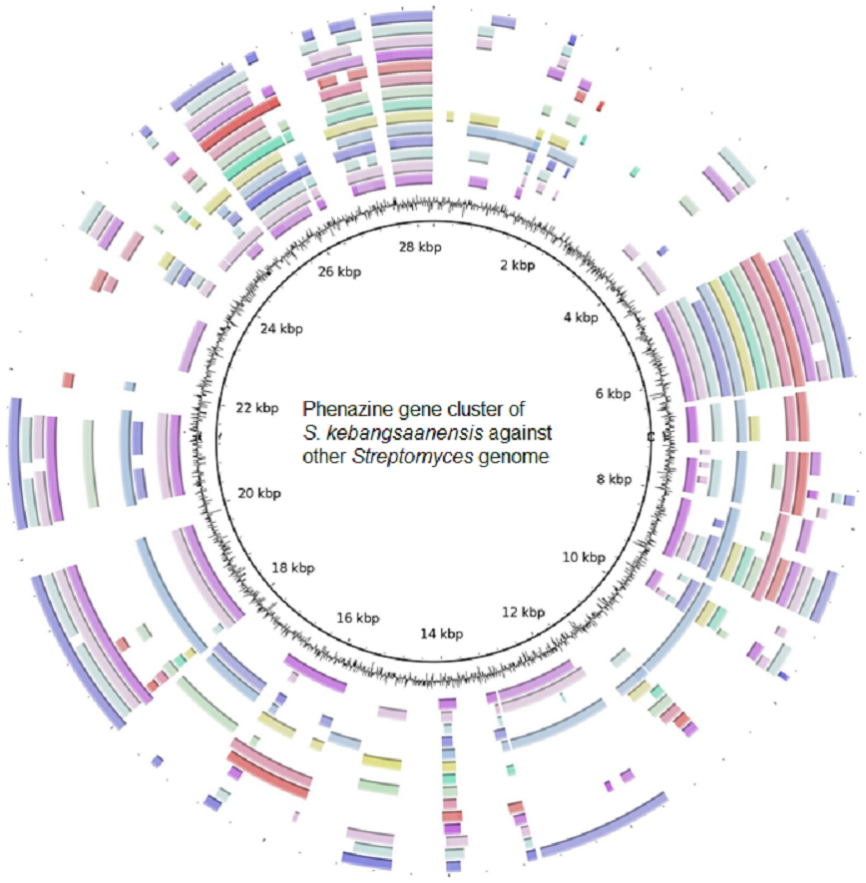
The putative phenazine gene clusters of *S. kebangsaanensis* BLASTed against other Streptomyces genomes. The first ring (black) represent the gene clusters of *S. kebangsaanensis* in base pairs as reference genes. The second ring is the GC content. The first coloured ring (purple) is *S. scabiei* 87.22, followed by *S. bingchenggensis, S. griseus, S. coelicolor*, *S. fulvissimus*, *S. venezuela, S*. *sp*. *PAMC* 26502, *S. hygroscopicus*, *S. albus* J1074, *S. sp. Sirex* AA-8, *S. davawensis, S. avertimilis*, *S. violaceusniger* and *S. cattleya*.

Furthermore, the *S. kebangsaanensis* phenazine gene cluster was also compared against 14 other complete genomes of *Streptomyces* to investigate whether the gene cluster was present in other *Streptomyces* as well (Fig. 4). It was clear that the phenazine gene cluster was mostly well conserved within all of these genomes, suggesting the existence of common genes and potentially pathways in the biosynthesis of phenazine, in particular of its backbone structure. However, several differences were also observed between these clusters suggesting that each species may produce different phenazine derivatives.

## Discussion

Endophytes are ubiquitous and are very likely to be found in all plant species (Rosenblueth & Martinez-Romero, 2006). In this mutual relationship, the host serves the microbes a protective niche for the microbes to live and in return, these microbes help the plant in their growth and development. Microbial secondary metabolites are low molecular weight, which usually produced during the late growth phase of microorganisms. They are not vital for the growth of the producing cultures but provide many survival functions in nature for the host (Ruiz et al., 2010). Actinomycete bacteria, especially those of the genus *Streptomyces*, are one of the most interesting bacteria that produce secondary metabolites with promising biological activity. These bacteria produce many classes of secondary metabolites with antibacteria, anticancer, antifungus, and antiinflammation activity, including polyletides and terpenes. For instant, analysis of secondary metabolites gene cluster in marine *Streptomyces* sp. MP131-18 showed that, six gene clusters with type 1 polyketide synthase, and five gene clusters for terpene biosynthesis were found within the genome of the bacteria (Paulus et al., 2017).

In this study, we found that *S. kebangsaanensis* produces a novel phenazine derivative termed HCPCA (Fig. 1), which is structurally dissimilar to any of the 11,609 phenazine structure compounds found in NCBI database (http://www.ncbi.nlm.nih.gov/). The closest related compounds was identified as saphenamycin (Fig. S2), which was isolated from *S. canaries* MG314-hF8 (Kitahara et al., 1982) and *S. antibiotics* (Geiger et al., 1988). However, several differences were noted between the functional groups of HCPCA compared to those of saphenamycin, such as the locations of hydroxyl, methyl, and phenol groups (Fig. S2). Saphenamycin displayed a broad spectrum of biological activities namely, antibacterial (Geiger et al., 1988), antitumour (Kitahara et al., 1982), and larvacidal activities as well as free radical scavenger (Laursen & Nielsen, 2004). Whereas, HCPCA exhibited strong antibacterial activity towards *Bacillus subtilis* ATCC 6633 (Sarmin, 2012).

Although *Streptomyces* are known to produce many phenazine derivatives only two gene clusters have been identified to date in *S. anulatus* (Saleh et al., 2009) and *S. cinnamonensis* (Haagen et al., 2006). Furthermore, out of 14 complete operons of phenazine biosynthesis documented in NCBI; nine were from *Pseudomonas* sp. and the remainder were from *Streptomyces* sp. (*S. anulatus*, *S. tendae*, *S. cinnamonensis*, *S. iakyrus*, and *S. griseoluteus*). Although the gene clusters share high similarities in operons, our study indicates that there were nine different genes from *S. kebangsaanensis* with no sharing homologous compared to other genome. The genes are; 3-oxoacyl-[acyl-carrier-protein] synthase III (gene 3268), N-acetyltransferase (gene 3274), polyketide cyclase (gene 3278), putative acyl-CoA synthetase (gene 3275), putative monooxygenase (gene 3276), while gene 3283, 3284, 3286, 3287 encoding for hemerythrin HHE, oxidoreductase, 3-oxoacyl-(Acyl-carrier-protein) synthase III, and 3-hydroxyacyl-CoA dehydrogenase, respectively. Given that HCPCA (Fig. 1) differs from all other known phenazine derivatives, unique gene sets or biochemical pathways may be required for its biosynthesis in *S. kebangsaanensis*. Hereby, we proposed a putative biosynthetic pathway of phenazine in this species by referring to the previously reported pathway (Blankenfeldt, 2013; Haagen et al., 2006; Mavrodi et al., 1998; McDonald et al., 2001) and the genome data that we obtained.

**Figure 5:**
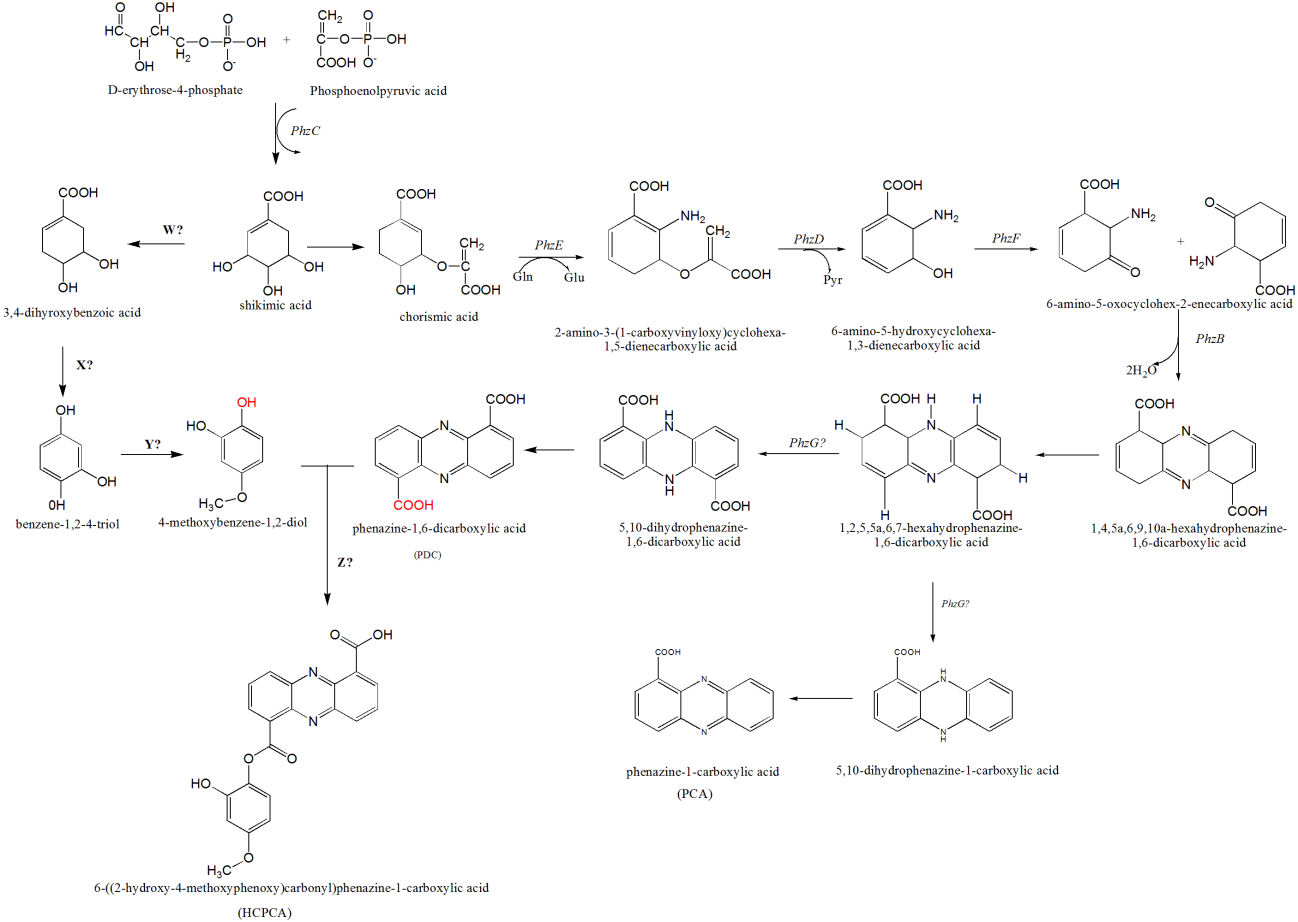
The proposed mechanism of phenazine biosynthesis in *S. kebangsaanensis.* The phenazine structure might be derived from the combination of two biosynthetic pathways which are phenazine-1,6-dicarboxylic acid (PDC) and 4-methoxybenzene-1,2-diol (MBD). These two pathways are originated from the shikimic acid pathway. Dehydration process between two functional groups of hydroxyl that present in both PDC and MBD will form 6-((2-hydroxy-4-methoxyphenoxy) carbonyl) phenazine-1-carboxylic acid (HCPCA).

Within *S. kebangsaanensis*, the HCPCA phenazine structure is hypothesised to be derived from the combination of two biosynthetic pathways, phenazine-1,6-dicarboxylic acid (PDC) and 4-methoxybenzene-1,2-diol (MBD) (Fig. 5). Both pathways are proposed to have originated from the shikimate pathway. Genes involved in the PDC and PCA pathways including *phz*E, *phz*D, *phz*F, *phz*B, and *phz*G, sequentially (Blankenfeldt, 2013; Mentel et al., 2009), all of which were found in the predicted phenazine cluster as proposed previously by McDonald et al. (2001), with the exception of *phz*G. *phz*G constitutes the final enzyme in PDC biosynthesis and converts 1,2,5,5a,6,7-hexahydrophenazine-1,6-dicarboxylic acid (HHPDC) to 5,10-dihydrophenazine-1,6-dicarboxylic acid (5,10-DHPDC), which is subsequently converted to PDC through a reduction process. Previous findings showed that *phz*G is similar to flavin mononucleotide-dependent pyridoxamine oxidases, which oxidize 6-amino-5-hydroxycyclohexane-1,3-dienecarboxylic acid to the respective 3-keto compound to form a tricyclic phenazine precursor (Mentel et al., 2009; Pierson et al., 1995). *phzG* was known to encode a protein exhibiting homodimeric flavin enzyme similar to pyridoxine-5′-phosphate oxidase. Notably, gene 3288 from our study was found to share 85% sequence identity to the LLM class F420-dependent oxidoreductase of *Streptomyces sp.* FxanaA7, a flavonoid cofactor dependent enzyme-like pyridoxine-5′-phosphate oxidase (Selengut & Haft, 2010). Therefore, it is possible that gene 3288 may assume the function of *phz*G to oxidize the HHPDC in the *S. kebangsaanensis* phenazine biosynthesis pathway (Fig. 5). Additionally, all the other eight genes mentioned previously may also individually or collectively play an important role in the modification of phenazine structure in *S. kebangsaanensis*. However, to confirm the proposed pathway, gene knock-out experiment will be needed to provide functional evidence for the genes that encoded in the cluster in phenazine biosynthesis pathway. Moreover, this will also help in the identification of the gene products that are currently unknown.

Conversely, the MBD pathway is proposed to branch off from chorismic acid to form 3,4-dihydoxybenzoic acid, followed by benzene-1,2-4-triol and finally MBD. However, specific genes (genes W, X, and Y) involved in the reactions of this pathway are still unknown. We speculate that gene W could be involved in the removal of one hydroxyl group from shikimic acid through a dehydration process, while gene X is proposed to be involved in dehydration at a carboxylic acid (COOH) functional group, and gene Y is involved in the addition of one methyl group (methylation) at a hydroxyl group. Furthermore, another gene (gene Z) that is involved in the dehydration process between the MBD hydroxyl and PDC carboxylic acid functional groups to form HCPCA is also unknown (red functional groups in Fig. 5). Additional studies utilizing genetic manipulation are likely required to verify the function of these individual genes in the biosynthetic pathway.

Whole genome sequencing of *S. kebangsaanensis* followed by bioinformatics analysis led to the discovery of different gene clusters believed to be involved in the production of numerous secondary metabolites. In particular, the *S. kebangsaanensis* genome was suggested to comprise a linear structure which was found in other species of *Streptomyces*, i.e., *S. lividans* (Lin et al., 1993) and *S. coelicolor* (Bentley et al., 2002). Nevertheless, the *S. kebangsaanensis* genome (8.3 Mbp) was noted to be shorter than other *Streptomyces* i.e., *S. coelicolor* (8.7 Mbp) (Bentley et al., 2002), *S. avermitilis* ATCC 31267 (8.7 Mbp) (Ōmura et al., 2001), and *S. griseus* IFO 13350 (8.5 Mbp) (Ohnishi et al., 2008) (Table 2).

**Figure 6:**
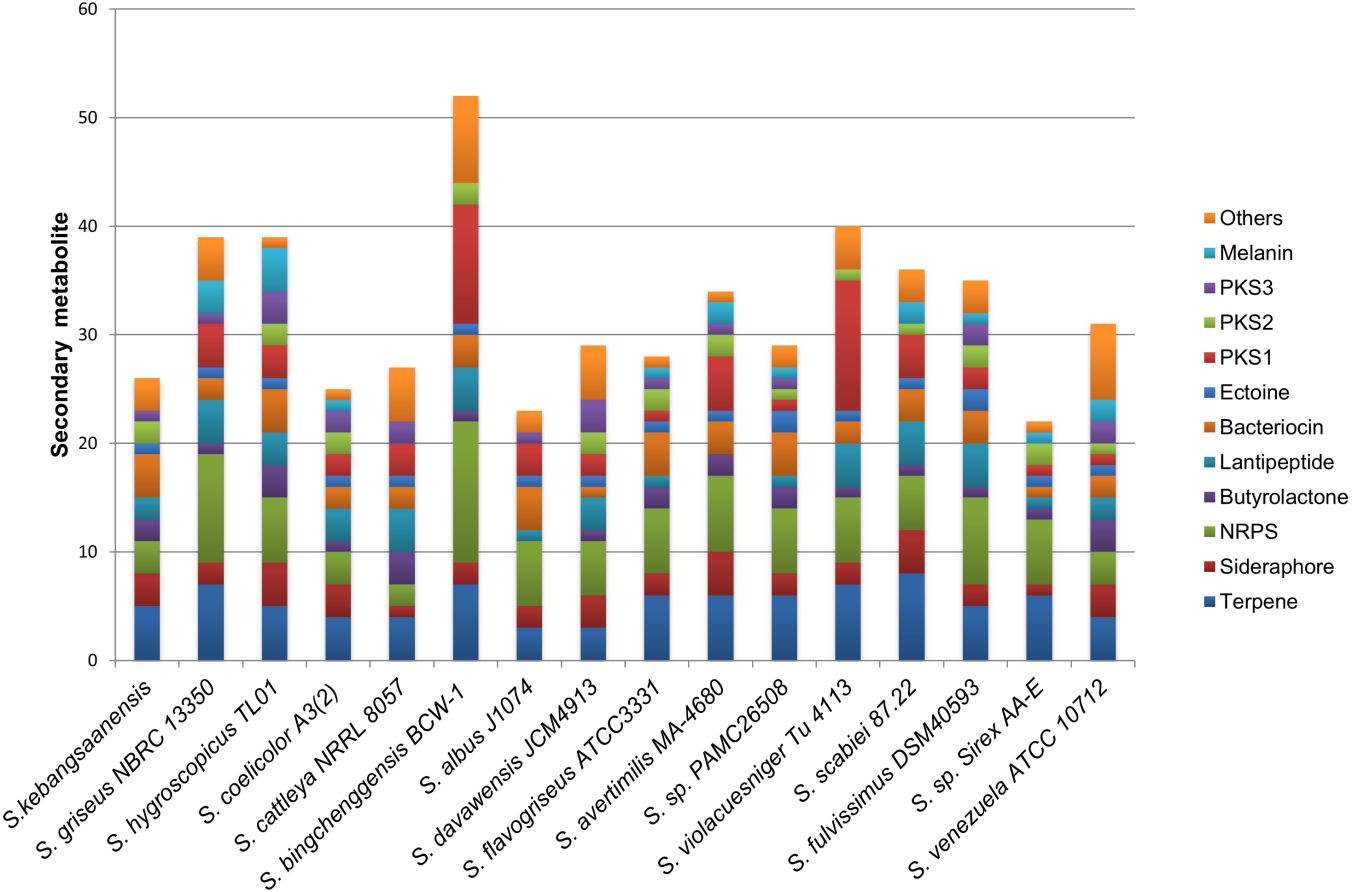
The comparison of secondary metabolite gene clusters between *S. kebangsaanensis* and the other genomes of *Streptomyces*. Each color in the cluster represents a different class or type of secondary metabolites or antibiotic. All *Streptomyces* including *S. kebangsaanensis* contain at least more than 20 gene clusters of secondary metabolite that may produce diverse bioactive compounds.

The genomic data also showed the presence of 24 biosynthetic gene clusters potentially involved in the production of secondary metabolites (Fig. 6). These gene clusters were comparable to those in other *Streptomyces* sp. such as *S. coelicolor*, *S. cattleya* NRRL 8057, and *S. flavogriseus*, which have 25, 27, and 28 gene clusters, respectively. As these *Streptomyces* come from similar genera, almost all genomes contained the same classes of secondary metabolite gene clusters such as terpenes, siderophores, NRPSs, butyrolactones, lantipeptides, PKSs, and melanins.

The production of different types of terpenes by *S. kebangsaanensis* was predicted based on the presence of four gene clusters. It is worth noting that, the anticancer drug paclitaxel (Taxol^®^) and the antimalarial drug artemisinin are among several terpenes with established medical applications (Paddon & Keasling, 2014). Terpene backbones are synthesized by two enzymes: isopentenyl-diphosphate and dimethylallyltransferase. The genes encoding these enzymes were also found to be present in *S. kebangsaanensis* (Fig. 3). Further analysis revealed the possibility of one of the terpene biosynthesis gene clusters being involved in producing an albaflavenone compound (with 50% identity to *S. viridochromogenes* DSM 40736). Albaflavenone is a novel sesquiterpene antibiotic first isolated from *S. coelicolor* that belongs to the phylum of actinobacteria (Zhao et al., 2008). Subsequently, genes encoding this metabolite including terpene synthases were found to be ubiquitous in bacteria, especially among *Streptomyces* (Yamada et al., 2015).

Siderophore biosynthetic genes were also predicted from the genome of *S. kebangsaanensis*. Over 10 distinct species of *Streptomyces* have been identified thus far to have the capability to produce desferrioxamine siderophores, such as desferrioxamine G, B, and E (Challis & Hopwood, 2003; Wang et al., 2014). In particular, our study pointed to the presence of one biosynthesis gene cluster involved in the production of desferrioxamine B (Fig. 3). The potential of its therapeutic application is reflected by the use of *S. pilosus* derived Desferrioxamine B, used for the treatment of iron intoxication (Nakouti, Sihanonth & Hobbs, 2012) and *Plasmodium falciparum* infection (Miethke & Marahiel, 2007). Furthermore, siderophores produced by endophytes have previously been given more attention due to their role in controlling soil borne plant pathogens (Loper & Buyer, 1991). For example, siderophores isolated from the endophyte *Streptomyces* sp. strain S96 were involved in inhibition of *Fusarium oxysporum* f. sp. cubense while also showed plant growth-promoting property (Cao et al., 2005). However, some siderophores from actinobacteria are also known to carry Fe molecule to *Rhizobium* including *Streptomyces lydicus* WYEC108, which colonizes roots and affects the nodulation of pear tree roots (Tokala et al., 2002). Therefore, siderophore biosynthetic gene clusters that are present in *S. kebangsaanensis* might hold key information pertaining to the growth promotion and inhibition of plant pathogens as well as towards its own survival in the plant.

In addition, all *Streptomyces* genomes have been shown to carry a single ectoine biosynthesis gene cluster (Fig. 6). In the *S. kebangsaanensis* genome, this biosynthesis gene cluster is located at scaffold 167 with a length of 10,408 bp (Fig. 3). The genes involved are ectoine/hydroxyectoine ABC transporter, L-ectoine synthase, and a putative ectoine hydroxylase, which pointed to the presence of the conventional route of ectoine production in *S. kebangsaanensis* (Pastor et al., 2010). Ectoine comprises one of the most extensively found compatible solutes throughout different halotolerant and halophilic microorganisms including actinobacteria from the *Brevibacterium* and *Streptomyces* genera (Pastor et al., 2010). Despite living in high ionic and hyperosmotic habitats, halophilic microorganisms are able to maintain proper osmotic balance to prevent cell leakage (Roeßler & Müller, 2001). Thus, the discovery of ectoines in nature may indicate significant applications including as protective agents for cellular components, in addition to their potential therapeutic uses (Pastor et al., 2010).

Furthermore, the genomic analysis also revealed that *S. kebangsaanensis* carries two biosynthetic gene clusters that are important in producing different types of antibiotics such as PKS Type II, as well as one cluster of PKS Type III and three clusters of NRPS biosynthetic genes (Fig. 3). PKS and NRPS comprise two classes of natural products with valuable biological activities (antimicrobial, antifungal, antiparasitic, antitumour, and cholesterol lowering agents as well as immunosuppressive agents), which are found mainly in bacteria (Du & Lou, 2010). The presence of PKS and NRPS are also common in other bacteria such as *S. coelicolor* (Bentley et al., 2002) and *S. avermitilis* (Ōmura et al., 2001).

Finally, *S. kebangsaanensis* might also produce different types of bacteriocin. For example, an informatipeptin pathway has been predicted in *S. kebangsaanensis* based on *S. gancidicus* BKS 13-15 and *S. prunicolor* NBRC 13075 gene clusters (Fig. 3). Bacteriocin has been isolated from most bacteria and archaea, each of which exhibited different structure, size, and mode of action as well as mechanism (Farris et al., 2011; Nes, Yoon & Diep, 2007). The presence of bacteriocin genes in *S. kebangsaanensis* in different scaffolds thus suggests the potential for this strain to produce different types of bacteriocin.

Overall, the genome of *S. kebangsaanensis* has revealed its potential for producing bioactive metabolites based on the 24 identified biosynthetic gene clusters. Therefore, future studies should be focused on specific metabolite identification and purification to shed light on new bioactive molecule discovery.

## Conclusion

*S. kebangsaanensis* represents a new endophyte that produces a novel compound, HCPCA. This structure has been elucidated using NMR and its novelty was demonstrated by structural comparison. Subsequently, genome sequencing of *S. kebangsaanensis* allowed the proposal of the phenazine biosynthetic pathway for this organism. We also identified several genes that are unique to *S. kebangsaanensis* in the phenazine cluster, which might be involved in the biosynthesis of HCPCA. The genome sequence also revealed numerous secondary metabolite gene clusters in *S. kebangsaanensis*, further analysis of which may lead to new and potentially bioactive secondary metabolites/antibiotics.

##  Supplemental Information

10.7717/peerj.3738/supp-1 Figure S1 Flow chart of the isolation of an active compound AF53611 (later recognised as HCPCA (6-((2-hydroxy-4-metoxyphenoxy) carbonyl) phenazine-1-carboxylic acid)) from *S. kebangsaanensis* crude extract

10.7717/peerj.3738/supp-2 Figure S2 The 6-((2-hidroxy-4-metoxyphenoxy)carbonyl)phenazine-1-carboxylic acid) (HCPCA) (1a) isolated from *S. kebangsaanensis* has a quite similar structure to saphenamycin (1b) isolated from *S. canaries* MG314-hF8 and *S. antibioticus*

10.7717/peerj.3738/supp-3 Figure S3 Neighbour-joining tree showing the relationship of *Streptomyces kebangsaanensis* based on full 16S rRNA gene sequence (1599 nt) with *Microbispora corollina* D65^T^ acts as the outgroup

10.7717/peerj.3738/supp-4 Table S1 Antibiotic resistance profile of strain SUK 12

10.7717/peerj.3738/supp-5 Table S2 Gradient elution step used in HPLC

10.7717/peerj.3738/supp-6 Table S3 NMR data ^1^H and ^13^C for compound AF53611

## Additional Information and Declarations

### DNA Deposition

The following information was supplied regarding the deposition of DNA sequences: DDBJ/EMBL/GenBank under BioProject PRJNA269542 and BioSample SAMN03254380.
