# Supplementary material for: Genomic characterization of a new endophytic Streptomyces kebangsaanensis identifies biosynthetic pathway gene clusters for novel phenazine antibiotic production
Source: PeerJ. 2017 Nov 29;5:e3738. doi: 10.7717/peerj.3738 (PMC5712208; doi:10.7717/peerj.3738)
Supplement: Figure S1 [file peerj-05-3738-s001.docx]

**Acetone crude extracts** (32.15 g)


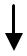


**Vacuum Liquid Chromatography (VLC)**

hexane:chloroform (8:2, 6:4); 100% chloroform; 100% methanol


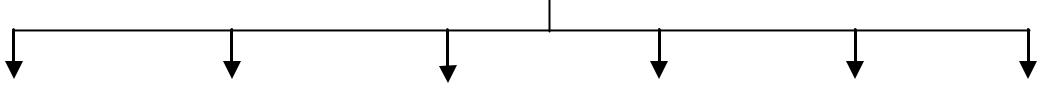


| F1 | F2 | F3 | F4 | **F5** | F6 |
| --- | --- | --- | --- | --- | --- |
| (2.85 g) | (2.56 g) | (2.69 g) | (3.22 g) | (2.01 g) | (18.58 g) |
|  |  |  |  |  |  |


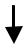


**Radial Chromatography (RC)** hexane:chloroform (9:1)


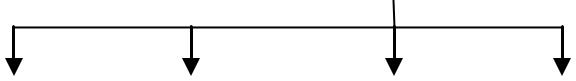


|  |  |  |  |  |  | F1 | |  | F2 |  | **F3** | |  |  |  | F4 | |
| --- | --- | --- | --- | --- | --- | --- | --- | --- | --- | --- | --- | --- | --- | --- | --- | --- | --- |
|  |  |  |  |  |  | (0.31 g) | |  | (0.41 g) |  | (0.52g) | | |  |  | (0.76 g) | |
|  |  |  |  |  |  |  |  |  |  |  |  |  | | |  |  |  |
|  |  |  |  |  |  |  |  |  |  | **RC** | | | | | | | |
|  |  |  |  |  |  |  |  |  |  | hexane:chloroform (2:8) | | | | | | | |
|  |  |  |  |  |  |  |  |  |  |  |  |  |  |  |  |  |  |
|  |  |  |  |  |  |  |  |  |  |  |  |  |  |  |  |  |  |
| F1 | F2 | | F3 | | F4 | | |  | F5 | F6 | |  |  | **F7** | | |  |
| (0.07 g) | (0.12 g) | | (0.03 g) | | (0.11 g) | | | (0.07 g) (0.08 g) | | | |  | (0.04 g) | | | |  |
|  |  |  |  |  |  |  |  |  |  |  | |  | | | |  |  |
|  |  |  |  |  |  |  |  |  |  | **RC** | | | | | | | |
|  |  |  |  |  |  |  |  |  | hexane:chloroform:methanol (7:2:1) | | | | | | | | |
|  |  |  |  |  |  |  |  |  |  |  |  |  |  |  |  |  |  |
|  |  |  |  |  |  |  |  |  |  |  |  |  |  |  |  |  |  |
|  |  |  |  |  |  | **F1** | |  | F2 |  | F3 | |  |  |  | F4 | |
|  |  |  |  |  |  | (19 mg) | | | (11 mg) | (29 mg) | | |  | (30 mg) | | | |
|  |  |  | **Preparative TLC** | | | | | | | | | |  |  |  |  |  |
|  |  |  |  |  | Hexane:ethyl acetate (3:7) | | | | |  |  |  |  |  |  |  |  |
|  |  |  |  |  |  |  |  |  |  |  |  |  |  |  |  |  |  |
|  |  |  |  |  |  |  |  |  |  |  |  |  |  |  |  |  |  |
|  |  |  | **F1** |  | F2 | | |  | F3 |  | F4 | |  |  |  |  |  |
|  |  |  | (0.8 mg) |  | (5.4 mg) | | |  | (7.1 mg) | (5.6 mg) | | | |  |  |  |  |


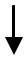

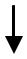

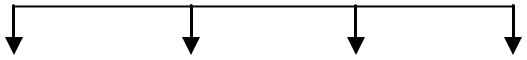

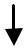

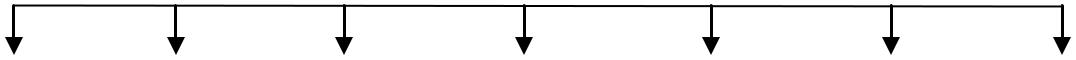

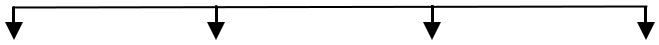

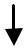


**High Performance Liquid Chromatography (HPLC)**

Gradient; methanol:water


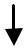


**AF53611**

(0.5 mg)

Figure S1. Flow chart of the isolation of an active compound AF53611 (later recognised as HCPCA (6-((2-hydroxy-4-metoxyphenoxy) carbonyl) phenazine-1-carboxylic acid)) from *S. kebangsaanensis* crude extract.
