# Supplementary material for: Genomic characterization of a new endophytic Streptomyces kebangsaanensis identifies biosynthetic pathway gene clusters for novel phenazine antibiotic production
Source: PeerJ. 2017 Nov 29;5:e3738. doi: 10.7717/peerj.3738 (PMC5712208; doi:10.7717/peerj.3738)
Supplement: Figure S2 [file peerj-05-3738-s002.docx]

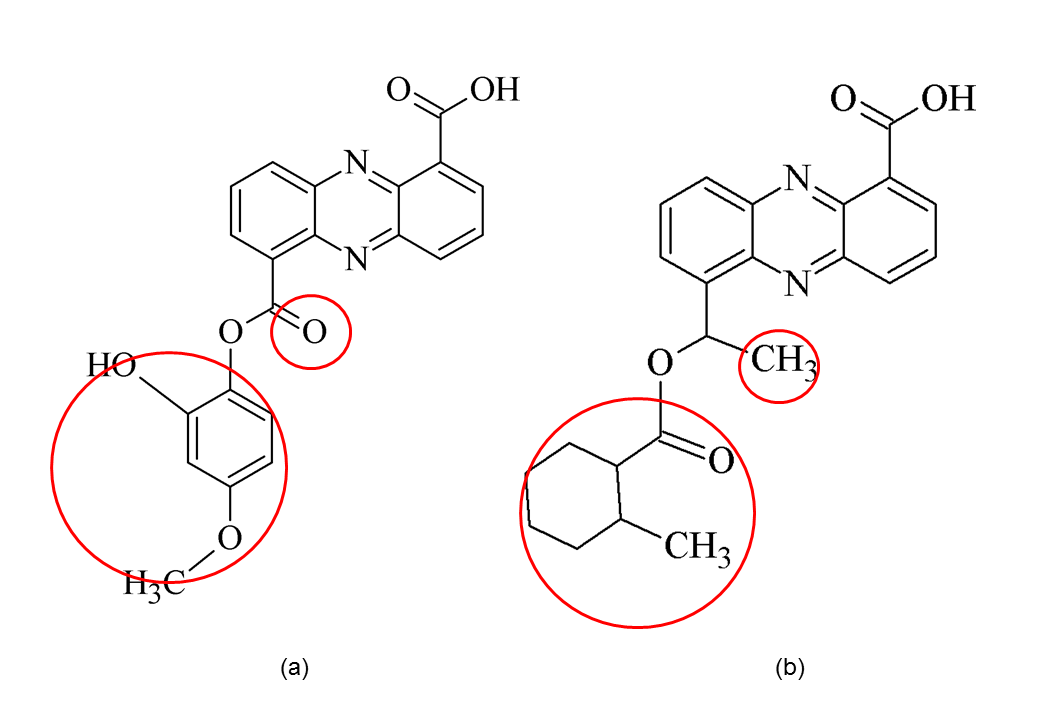


Figure S2. The 6-((2-hidroxy-4-metoxyphenoxy)carbonyl)phenazine-1-carboxylic acid) (HCPCA) (1a) isolated from *S. kebangsaanensis* has a quite similar structure to saphenamycin (1b) isolated from *S. canaries* MG314-hF8 and *S. antibioticus*. The red circle showed the difference between functional groups of these compounds.
