# Supplementary material for: Genomic characterization of a new endophytic Streptomyces kebangsaanensis identifies biosynthetic pathway gene clusters for novel phenazine antibiotic production
Source: PeerJ. 2017 Nov 29;5:e3738. doi: 10.7717/peerj.3738 (PMC5712208; doi:10.7717/peerj.3738)
Supplement: Figure S3 [file peerj-05-3738-s003.docx]

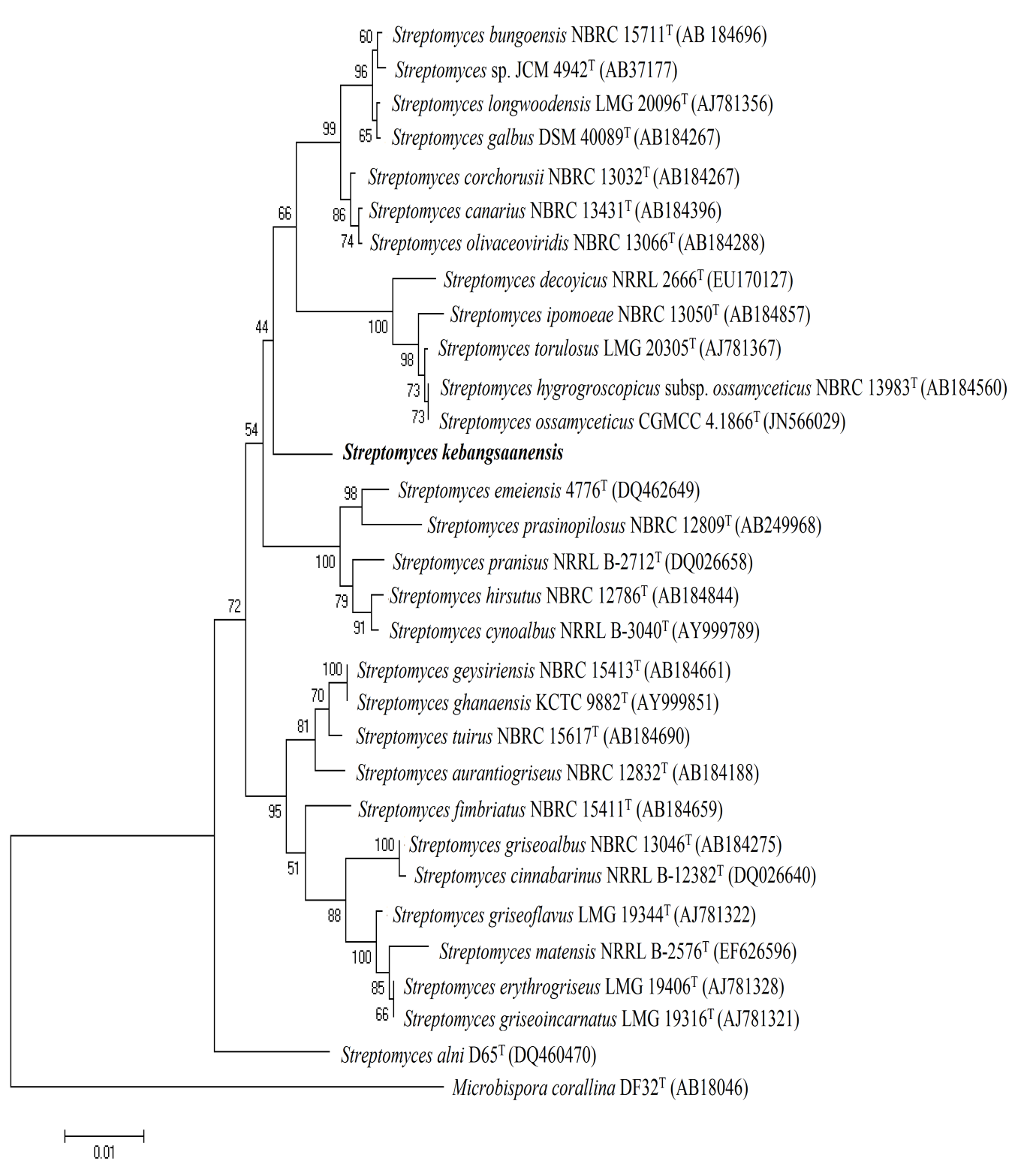


Figure S3. Neighbour-joining tree showing the relationship of *Streptomyces kebangsaanensis* based on full 16S rRNA gene sequence (1599 nt) with *Microbispora corollina* D65^T^ acts as the outgroup. Asterisks indicate branches of the tree that were also recovered by using maximum-parsimony and maximum-likelihood algorithms. Numbers at the nodes indicate levels of bootstrap support based on 1000 resampled datasets. Bar represents 0.01 changes per nucleotide.
