# Supplementary material for: Genomic characterization of a new endophytic Streptomyces kebangsaanensis identifies biosynthetic pathway gene clusters for novel phenazine antibiotic production
Source: PeerJ. 2017 Nov 29;5:e3738. doi: 10.7717/peerj.3738 (PMC5712208; doi:10.7717/peerj.3738)
Supplement: Table S1 [file peerj-05-3738-s004.docx]

Table S1 Antibiotic resistance profile of strain SUK 12

| **Test** | **Result** |
| --- | --- |
| Antibiotic resistance (µg): |  |
| vancomycin (30) | S (40)* |
| gentamicin (10) | S (22) |
| ampicillin (10) | R |
| penicillin G (10) | R |
| amphotericin B (50) | R |
| tetracyclin (125) | S (40) |
| streptomycin (50) | S (16) |
| methicillin (50) | R |
| cyclohexamide (50) | R |
| oxacillin (50) | R |
| nystatin (50) | R |
| nalidixic acid (50) | R |

* Number in bracket shows zone of inhibition in millimeter unit.

S = sensitive

R =Resistance
