# Supplementary material for: Genomic characterization of a new endophytic Streptomyces kebangsaanensis identifies biosynthetic pathway gene clusters for novel phenazine antibiotic production
Source: PeerJ. 2017 Nov 29;5:e3738. doi: 10.7717/peerj.3738 (PMC5712208; doi:10.7717/peerj.3738)
Supplement: Table S2 [file peerj-05-3738-s005.docx]

Table S2 Gradient elution step used in HPLC

| Bil | Time (min) | Flow rate (ml/min) | Water (%) | Methanol (%) |
| --- | --- | --- | --- | --- |
| 1. | 0.00 | 1.00 | 95 | 5 |
| 2. | 1.00 | 1.00 | 95 | 5 |
| 3. | 21.00 | 1.00 | 0 | 100 |
| 4. | 25.00 | 1.00 | 0 | 100 |
| 5. | 28.00 | 1.00 | 95 | 5 |
| 6. | 30.00 | 1.00 | 95 | 5 |
