# Supplementary material for: Genomic characterization of a new endophytic Streptomyces kebangsaanensis identifies biosynthetic pathway gene clusters for novel phenazine antibiotic production
Source: PeerJ. 2017 Nov 29;5:e3738. doi: 10.7717/peerj.3738 (PMC5712208; doi:10.7717/peerj.3738)
Supplement: Table S3 [file peerj-05-3738-s006.docx]

Table S3 NMR data ^1^H and ^13^C for compound AF53611

| **Atom C** | **AF53611 (^1^H, δ, MeOD)** | **AF53611(^13^C, δ, MeOD)** |
| --- | --- | --- |
| C_1_ (C) | - | 126.22 |
| C_2_ (CH) | 8.47, dd, *J*= 8.64, 1.5 | 133.68 |
| C_3_ (CH) | 8.08, m | 129.83 |
| C_4_ (CH) | 8.86, dd, *J*= 7.08, 1.5 | 134.94 |
| C_4a_ (C) | - | 142.50 |
| C_5a_ (C) | - | 143.06 |
| C_6_ (C) | - | 129.02 |
| C_7_ (CH) | 8.03, m | 131.51 |
| C_8_ (CH) | 8.06, m | 131.96 |
| C_9_ (CH) | 8.38, m | 128.91 |
| C_9a_ (C) | - | 140.49 |
| C_10a_ (C) | - | 140.18 |
| C_1’_ (C=OOH) | 15.61, s | 163.90 |
| C_2’_ (C=OO) | - | 161.80 |
| C_3’_ (C) | - | 138.36 |
| C_4’_ (C-OH) | 7.14, m | 123.61 |
| C_5’_ (CH) | 7.40, m | 118.84 |
| C_6’_ (C-O) | - | 147.29 |
| C_7’_ (CH) | 7.22, m | 123.61 |
| C_8’_(CH) | 7.45, m | 124.52 |
| C_9’_ (CH3) | 1.34, m | 29.36 |

* δ_H_, ppm, 600 MHz; δ_C_, ppm, 150 MHz; *J* couplings, *J*, Hz
